# Deer antler extract promotes tibia fracture healing in mice by activating BMP-2/SMAD4 signaling pathway

**DOI:** 10.1186/s13018-022-03364-2

**Published:** 2022-10-28

**Authors:** Jianyu Wang, Yuchi Wei, Zhenwei Zhou, Jie Yang, Yuyan Jia, Hailong Wu, Haisi Dong, Xiangyang Leng

**Affiliations:** 1 College of Traditional Chinese Medicine, Changchun University of Chinese Medicine, Changchun, 130117 Jilin Province China; 2 Jilin Ginseng Academy, Changchun University of Chinese Medicine, Changchun, 130117 Jilin Province China

**Keywords:** Deer antler extract, Fracture healing, BMP-2/SMAD4 signaling pathway, MC3T3-E1 cells

## Abstract

**Background:**

Deer antler is a traditional Chinese medicine with the function of tonifying kidney and strengthening bone, which is often used to treat orthopedic diseases.

**Methods:**

Eight-week-old C57BL/6 mice were used as the fixation model of open tibial fracture with intramedullary nail. The mice were treated with deer antler extract (DAE) or PBS by oral gavage once daily. The tibial fracture samples were collected and performed to the tissue analysis, including X-ray, micro-CT, histology, qRT-PCR, immunohistochemistry. MC3T3-E1 cells were used to detect the effect of deer antler extract on ability of cell proliferation and migration by CCK-8 assay and cell scratch test.

**Results:**

Imaging and micro-CT showed that DAE could promote the healing of tibial fracture in mice, and histological analysis showed that DAE could promote the transformation of cartilage callus to bone callus in fracture area. The results of qRT-PCR and immunohistochemistry showed that DAE could promote intrachondral ossification in fracture zone and the mechanism of promoting fracture healing may be related to the activation of BMP-2/SMAD4 signaling pathway. In the cytological experiment of DAE, it can be found that DAE promoted the proliferation of MC3T3-E1 cells and the migration of MC3T3-E1 cells at a certain concentration, which is also related to the promotion of fracture healing by DAE.

**Conclusion:**

DAE can promote fracture healing by activating BMP-2/SMAD4 signaling pathway. DAE has the potential to be used in clinic as an important means of promoting fracture healing.

## Background

Tibial fractures are one of the common types of fractures in bone trauma. Partial or complete fracture of bone brings great pain to patients. The tibial fractures account for 4% of limb fractures [1]. In clinical practice, the incidence of nonunion of tibial shaft fractures is 5%, and the incidence of delayed union of open tibial fractures is 6.80%. The incidence of delayed healing in the middle third of tibia is as high as 92.40%, which brings many difficulties to clinical treatment [2–7].

Fracture healing process is divided into intramembranous ossification and intrachondral ossification. Intramembrane ossification is a process of the direct differentiation of bone marrow stromal cells into osteoblasts; intrachondral ossification refers to the differentiation of bone marrow stromal cells into chondrocytes, which differentiate, proliferate, mature, hypertrophy and eventually replaced by bone [8]. The process of fracture healing originates from the formation of hematoma and inflammatory reaction in the broken end of the fracture and surrounding soft tissue. Inflammatory cells recruit local chemokines around the wound. The debridement led to the proliferation of mesenchymal stem cells in the bone marrow cavity at the fracture site and around the periosteum and periosteum, and the undifferentiated mesenchymal cells in the adjacent soft tissue were continuously supplemented. If the fracture is absolutely stable, the fracture is healed by simple intramembranous ossification. However, in most cases, the fracture end will appear certain instability, which will lead to intrachondral bone healing.

Chondrocytes and fibroblasts gradually differentiated under the induction of the interaction of different cytokines and growth hormones, forming granulation tissue and cartilage callus at the fracture healing site, and gradually stabilizing the fracture site. The callus stage is the most active stage of osteogenesis. BMPs play a very important role in the differentiation, proliferation and synthesis of extracellular matrix of osteoblasts. Extracellular matrix develops into callus through vascularization and mineralization to produce cancellous bone. Subsequently, it is a series of osteogenic processes dominated by osteoblasts and osteoclasts. Finally, through bone reconstruction and shaping, woven bone gradually forms mature cortical and cancellous lamellar bone to achieve bone healing without fibrous scar [9]. In fact, a variety of growth factors and signaling pathways may be activated during fracture healing. Among them, bone morphogenetic proteins (BMPs) are the most closely related to induce osteogenesis, bone formation and adult bone repair during normal embryonic period [10]. BMP/SMAD signaling pathway is one of the most important cellular signaling pathways during distraction osteogenesis [11, 12].

Deer antler is the immature horn of the unossified dense hairs of the sika deer or horse deer. As a kind of animal medicine, deer antler mainly contains protein polypeptide components, free amino acids, inorganic elements, carbohydrates, biogenic amines, steroids and lipids [13]. Among them, protein polypeptides are the most complex and have the most extensive effects, antler polypeptides and polysaccharides can promote the proliferation of osteoblasts and have a positive effect on regulating bone metabolism. The experiments showed that both antler collagen, antler polypeptide, Chinese herbal compound and Chinese patent medicine preparations could regulate bone metabolism, promote bone formation and inhibit bone loss [14]. However, the role and mechanism of promoting fracture healing are not clear. This experiment intends to do further research on this part and analyzes its mechanism of promoting fracture healing.

## Methods

### Materials

The deer antler used in this study was the same as the ones prepared as previously described [15]. DMEM high glucose medium (Sigma, USA); fetal bovine serum (Israel Biological Industries Corporation, USA); green/streptomycin double antibody (MedChemExpress company, USA); trypsin (MRC, USA); Alizarin Red dye (Beijing Solebo Technology Co., Ltd. China); CCK-8 kit (Beijing Boson Biotechnology Co., Ltd. China); primers (Jilin Province Kumei Biotechnology Co., Ltd. China); fluorescence quantitative PCR kit (BORI Doctor Technology (Beijing) Co., Ltd. China), CO2 cell culture box (Thermo Fisher Company, USA); freeze dryer (American LABCONCO company, USA); PCR instrument (Bio-Rad company, USA); ultra-clean workbench (Suzhou Purification Equipment Co., Ltd. China); injected Constant Temperature Tank (Shanghai Alan Instrument Co., Ltd. China), -80℃low temperature refrigerator (Haier Company, China); full wavelength microplate reader (Swiss TECAN company, China); optical microscope (Olympus, Japan); and table centrifuge (Germany Eppendorf company, Germany).

### Animals and model design

All experimental mice were C57BL/6 mice (*N* = 42) from Liaoning Changsheng Biotechnology Co., Ltd., fed by SPF Animal Center of Changchun University of Traditional Chinese Medicine (SYXK (Ji) 2018-0014). All experimental procedures were guided and permitted by the Animal Ethics Committee of Changchun University of Traditional Chinese Medicine (number: ccucm-2017-0015). Anesthesia was performed by intraperitoneal injection of pentobarbital sodium (60 mg/kg), and the fixation model of intramedullary nail for tibial fracture was performed after anesthesia. At the same time, all fracture animal models were divided into model group (*n* = 21) and DAE group (*n* = 21). Tissue samples were extracted from each group on the 7th, 14th, 21st and 28th day for qRT-PCR analysis (*n* = 3). Tissue samples were collected the 14th, 21st and 28th day for imaging and histological analysis (*n* = 3). Mice in DAE group were given DAE 4 mg/10 g/d by gavage according to body surface area normalization method. Mice in DAE group were given DAE 4 mg/10 g/d by gavage according to body surface area normalization method [16], and mice in model group were given PBS by gavage for comparison.

### X-ray filming

Place tibial fracture area in visual field. Determine whether the model is successful, and check the X-ray results on the 7th, 14th, 21st and 28th day to see the fracture healing.

### Micro-CT analysis

Tibia was taken on the 14th, 21st and 28th day, and metal needles were pulled out, using Skyscan 1174 micro-CT scanner software (voltage, 50 kv; current, 800 μA). The scanning resolution is 14.5 μ, and the field of view is 1304 × 1024. A total of 100 consecutive sections of the fracture area at different stages, including the bone marrow cavity with a thickness of 1.8 mm, were used for imaging the regions of interest in three-dimensional reconstruction. Callus tissue volume (TV), callus bone volume (BV), bone surface (BS), trabecular thickness (Tb.Th) and trabecular separation (Tb.Sp) were measured for quantitative analysis of bony callus.

### Histological analysis

On the 14th, 21st and 28th days after successful modeling, the callus and bone tissue in the fracture zone were taken, and the tissue in the fracture zone was fixed with 4% paraformaldehyde, followed by decalcification with 14% EDTA solution for 14 days and paraffin embedding. The slices with the thickness of 3 μm were cut and analyzed by saffron O fixation green staining.

### Quantitative real-time PCR analysis (qRT-PCR)

The expression levels of the identified DEGs were further validated by quantitative real-time PCR (qRT-PCR). The relative gene expression level was normalized to the mouse glyceraldehyde-3-phosphate dehydrogenase gene (Gapdh) following the 2-ΔΔCT method. Primers were synthesized by Jilin Kumei Biotechnology Co., Ltd. The sequence of primers is shown in Table 1.

**Table 1:** Primer sequence

Gene name		5ʹ - 3ʹ
GAPDH	Forward primer	CCTGCACCACCAACTGCTTA
	Reverse primer	GGCCATCCACAGTCTTCTGAG
RUNX2	Forward primer	CTGGCCTTCCACTCTCAGTAA
	Reverse primer	ACTGGCGGGGTGTAAGTAAAG
COL1A1	Forward primer	GTGGCGGTTATGACTTCAGC
	Reverse primer	TCACGAACCACGTTAGCATC
COL2A1	Forward primer	ACATAGGGCCTGTCTGCTTCTTGT
	Reverse primer	TGACTGCGGTTGGAAAGTGTTTGG
SOX9	Forward primer	TCCACGAAGGGTCTCTTCTC
	Reverse primer	AGGAAGCTGGCAGACCAGTA
COL10A1	Forward primer	TGGAAACCCGAGGTATGCTT
	Reverse primer	CATTGCATTGCACGTCATCG
ALP	Forward primer	GTATGGGCGTCTCCACAGTA
	Reverse primer	CTTCACGCCACACAAGTAGG
OPN	Forward primer	CAGCCATGAGTCAAGTCAGC
	Reverse primer	TGTGGCTGTGAAACTTGTGG
BMP-2	Forward primer	GAACCCAGGTGTCTCCAAGA
	Reverse primer	GTCTCTTGCAGCTGGACTTG
BMPR-2	Forward primer	GAACCCAGGTGTCTCCAAGA
	Reverse primer	GTCTCTTGCAGCTGGACTTG
SMAD4	Forward primer	ATTCCAGCCTCCCATTTCCA
	Reverse primer	TCCCGAAGGATCCACATAGC

### Immunohistochemical staining

The samples of bone and callus tissue were fixed, decalcified and embedded in paraffin for 7 μm sections, and immunohistochemical staining was performed. The following antibodies were used: BMP-2 (Servicebio GB12252, dilution 1:200), BMPR-2 (Servicebio GB111333, dilution 1:50), SMAD4 (Servicebio GB11174, dilution 1:50), RUNX2 (Servicebio GB13264, dilution 1:50) and horseradish peroxidase-conjugated streptavidin (Servicebio GB23301, dilution 1:200). Image analysis software was used to quantify the positive staining area.

### Cell viability assay and wound scratch test

MC3T3-E1 cell line was cultured in basic medium (DMEM medium + 10% fetal bovine serum + 1% penicillin/streptomycin double antibody), and the medium was changed every other day. The cells in logarithmic growth phase were used for this experiment. The cells were inoculated in a 96-well culture plate at a density of 3000 cells/100μL, with three replicate wells in each group. Different concentrations of deer antler extract were added. After 24 h of culture, 10 μL CCK-8 reagent was added to each well and incubated at 37 °C for 1 h. The absorbance at 450 nm was detected. For wound scratch test, with a marker pen in 6 holes behind the plate with a ruler to draw a uniform line, about every 1 cm draw one, each hole 3, inoculate 1 ml per well at a density of 500,000 cells ml^−1^. When the cell fusion rate reaches 100%, scratch with 1 ml gun head along the ruler, perpendicular to the transverse line, with moderate intensity. Use the same gun head between different holes to draw a straight line, and observe and confirm under the microscope. 2 ml PBS was added to each well, gently shake, wash twice, and remove the cells under scratch, according to the grouping which were added to the normal medium and drug-containing medium; photographs record the initial scratch state, placed in the incubator culture, respectively, after scratch and scratch 8, 16, 24 h, take photographs again, and the site should be the same as the first site. Observe cell migration.

### Statistical analysis

GraphPad Prism software was used for statistical analysis. The enumeration data were tested using the chi-square (*χ*^2^) test. The measurement data were first analyzed to confirm that normal data distribution was satisfied. If the normal distribution was satisfied, the *t* test was used. If the normal distribution was not satisfied, the rank-sum (*Z*) test was used. The test level *α* = 0.05 and *P* < 0.05 indicated that the difference was statistically significant.

## Results

Previous studies from our group have identified the components of the DAE by proteomics and liquid chromatography-mass spectrometry, and the results indicated that DAE included a variety of system functional proteins, such as antibacterial proteins, nervous system functional proteins, blood system functional proteins, energy synthase, cartilage and osteogenic development-related proteins. The proteins related to cartilage and bone development mainly include transmembrane glycoprotein NMB, peroxiredoxin-6 and S-adenosylmethionine synthase-5. At the same time, there are extracellular matrix proteins that promote cartilage ossification and participate in the process of endochondral ossification, mainly including pleiotrophin, periostin, alkaline phosphatase and biglycan. These proteins play an important role in regulating endochondral ossification, bone mineral density and bone tissue damage repair. Moreover, other cell tissues and organ development eggs contained in DAE play an important role in regulating cytoskeleton, tissue and organ development and cell differentiation, accelerating angiogenesis and regulating signal of bone cell function [17, 18].

**Fig. 1:**
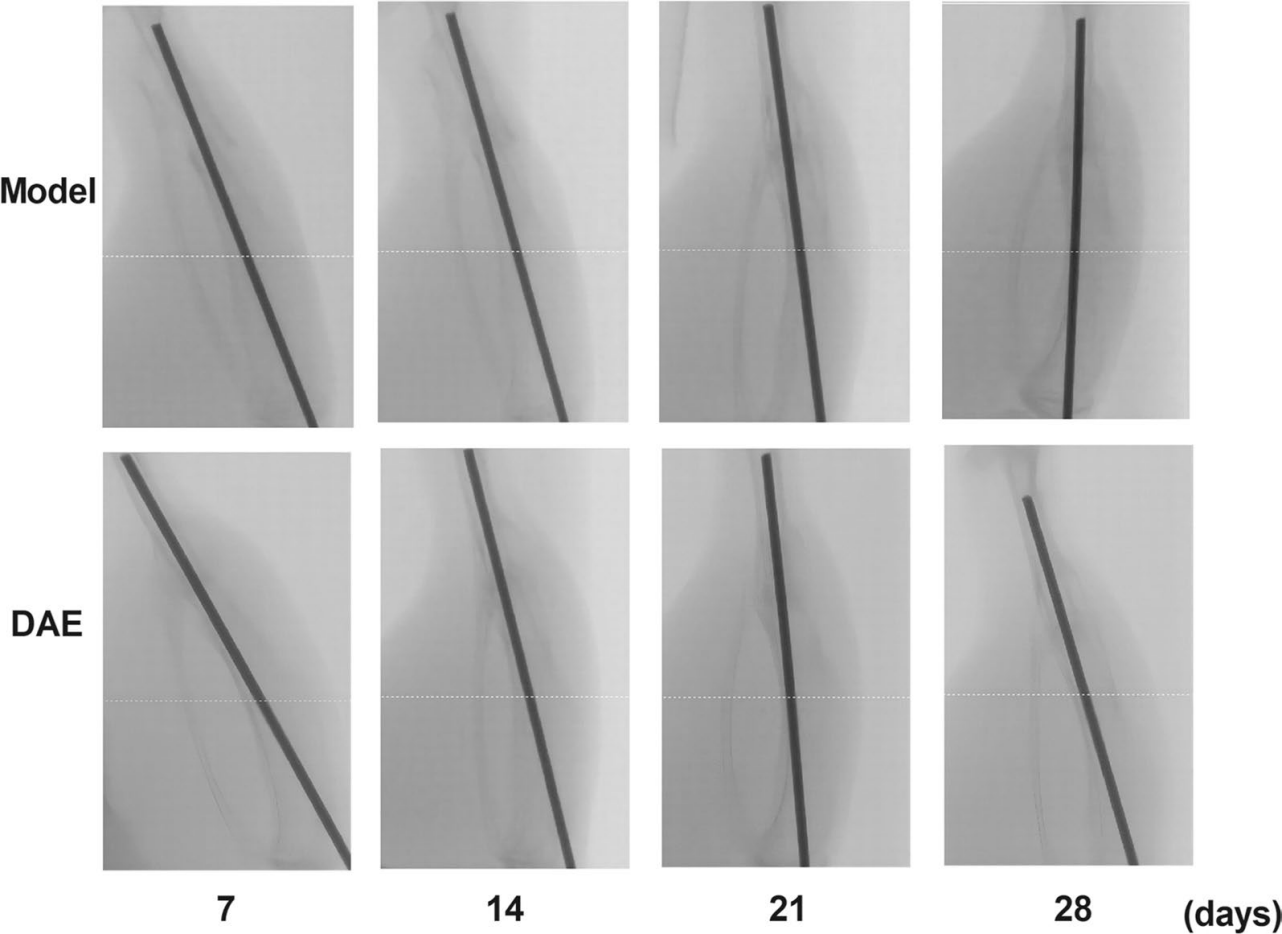
The X-ray manifestations of model group and DAE group at 7, 14, 21, 28 days after fracture. X-ray radiographic analysis showed that compared with the model group, the fracture line of the DAE treatment group appeared fuzzy callus on the 7th day, while the model group appeared this phenomenon on the 14th day, and the fracture healing of the DAE group was faster

In order to determine the effect of DAE on fracture healing, C57BL/6 mice were treated with intramedullary nail fixation model of tibial transverse fracture and DAE. X-ray filming radiological analysis showed that compared with model group, the fracture line of the DAE treatment group appeared fuzzy and bony callus on the 7th day, and the model group appeared this phenomenon on the 14th day, and the fracture line of the DAE treatment group almost disappeared on the 28th day. Although the fracture line of the model group was fuzzy, it was still clearer than that of the treatment group. In the DAE group, the callus and fracture morphology began to change on the 28th day. Compared with the 21st day of the same group, the callus was reduced and the fracture line was more blurred. Considering that it has entered the callus plastic stage, but the model group did not appear this phenomenon, which is one of the evidences that DAE promotes fracture healing and shortens fracture healing period (Fig. 1).

**Fig. 2:**
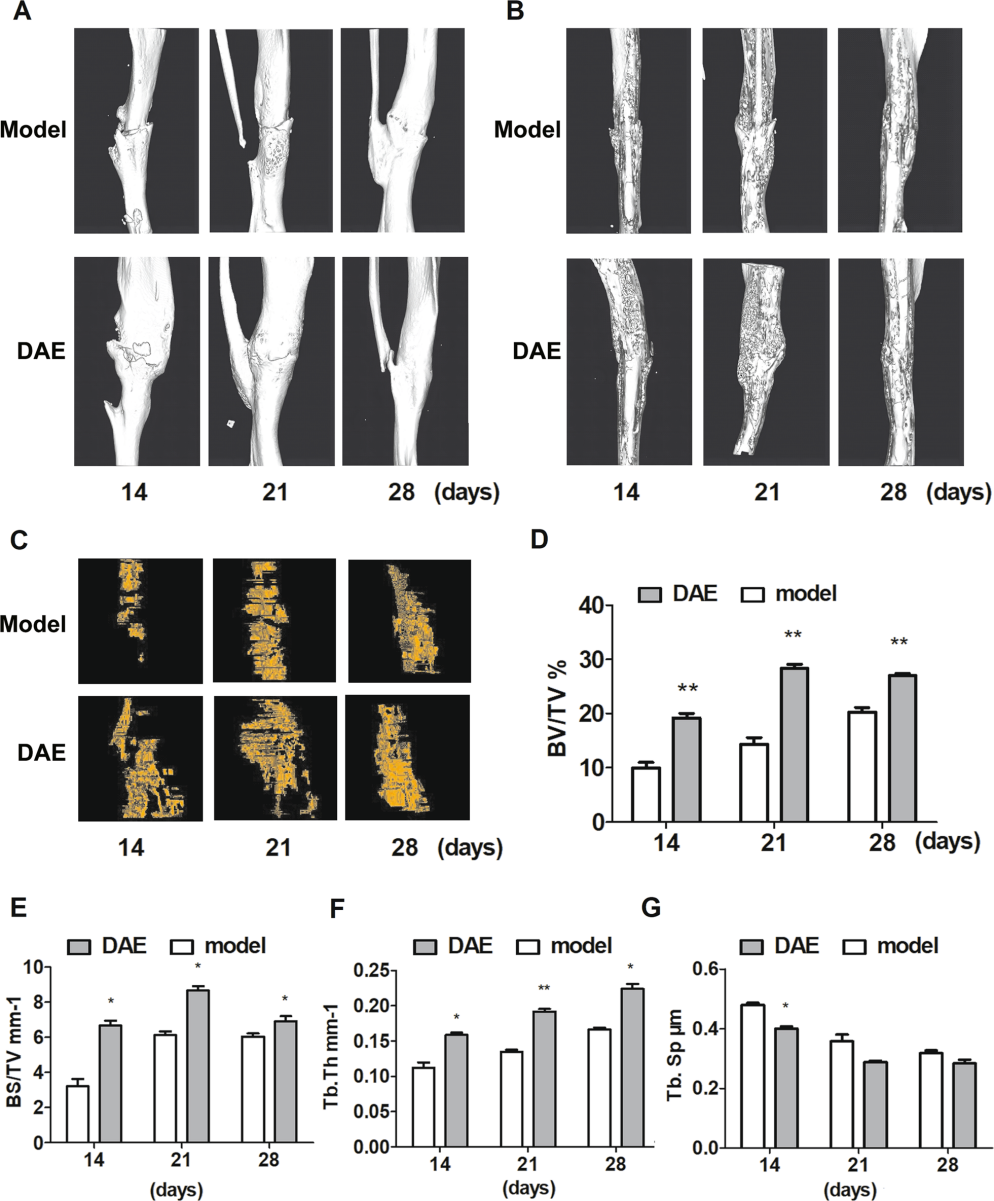
Microscopic CT analysis of fracture in model group and DAE group at 14, 21 and 28 days after fracture. **A** 3D reconstruction images representing the fracture zone of model group and DAE group. **B** 3D sectional images representing model group and DAE group. **C** 3D image display of new trabecular bone in fracture zone of model group and DAE group. **D** Bone volume-to-tissue volume ratio. **E** Bone surface-to-tissue volume ratio. **F** Bone surface-to-tissue volume ratio. **G** Trabecular thickness (Tb.Th). **H** Trabecular separation (Tb.Sp) (Model group sample data as control, *0.05 > *P* > 0.01; **0.01 > *P* > 0.001; ***P < 0.001; *n* = 3)

Micro-CT three-dimensional reconstruction analysis showed that compared with the model group, the fracture area of mice treated with DAE was smooth on the 28th day, and there was no obvious fracture trace. In the DAE group, the fracture area on the 28th day was thinner than that on the 21st day, which was closer to the normal bone tissue, suggesting that it had entered the callus shaping stage, which was consistent with the results of appeal X-ray (Fig. 2A). From the fracture zone reconstruction image, it can be seen that the fracture ends of the two groups were aligned and aligned well. On day 14 and 21, DAE group had better callus coverage and thicker callus than model group. At the 28th day, the callus in the model group was also covered well, but the callus in the DAE group was relatively reduced, the fracture traces were very vague, and the fracture was close to complete healing (Fig. 2B). Compared with the model group, the number of new bone trabecula in the fracture area of the mice treated with DAE increased significantly on the 14th and 21st day. In the DAE group, the range of new bone trabecula was smaller on the 28th day than on the 21st day, but the new bone trabecula combined more closely, which continued to confirm that it had entered the stage of fracture remodeling, and the model group did not appear this phenomenon (Fig. 2C). BV/TV and BS/TV can show the amount of bone in the fracture area, and are positively correlated with the amount of bone. Tb.Th represents the thickness of trabecular bone, which is positively correlated with bone mass. Tb.Sp represents the degree of trabecular separation, which is negatively correlated with bone mass. Compared with the model group, the BV/TV, BS/TV and Tb.Th of mice treated with DAE increased significantly on the 14th, 21st and 28th day, and Tb.Sp decreased significantly on the 14th day (Fig. 2D–G). Tb.Sp in DAE group decreased on day 21 and 28, but the difference was not statistically significant.

**Fig. 3:**
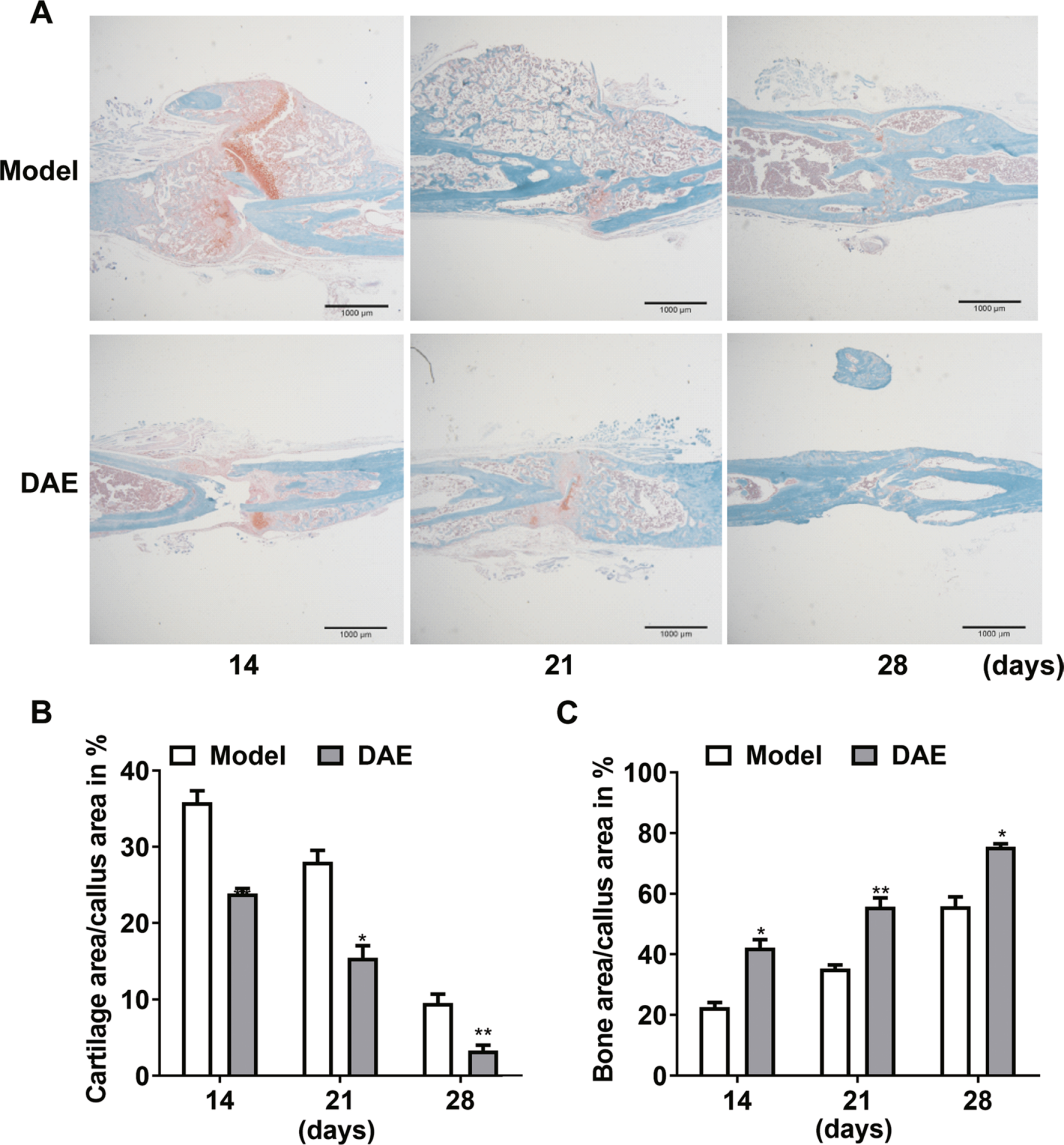
Histomorphometric analysis of fracture zone in model group and DAE group at 14, 21, 28 days after fracture. **A** Comparison of safranin O staining in fracture zone between model group and DAE group. **B** Relative cartilage area in the periosteal fracture callus at 14, 21 and 28 days after fracture. **C** Relative bone area in the periosteal fracture callus at 14, 21 and 28 days after fracture. (Model group sample data as control, *0.05 > *P* > 0.01; **0.01 > *P* > 0.001; ***P < 0.001; *n* = 3)

Saffron O fixation green staining showed that the fracture healing of the antler extract treatment group was faster (Fig. 3A). Histological and histomorphological analysis showed that the fracture ends of the two groups were in good alignment and alignment, and the fracture area had healing performance. The tibial fracture model was successful. The proportion of cartilage in the fracture area of DAE treated mice was significantly reduced on the 14th, 21st and 28th day, indicating that the cartilage callus gradually ossification and cartilage volume gradually reduced during fracture healing (Fig. 3B). On the 14th, 21st and 28th day, the ossification of fracture zone in mice treated with DAE was more obvious, and the proportion of bone tissue in fracture zone was significantly increased, especially on the 28th day, the cartilage in fracture zone was almost completely ossification, and the proportion of cartilage was less than 10% (Fig. 3C). According to the healing mode of bone formation in fracture cartilage, it showed that the DAE group almost completely healed, and the treatment of DAE accurately shortened the healing time of fracture.

**Fig. 4:**
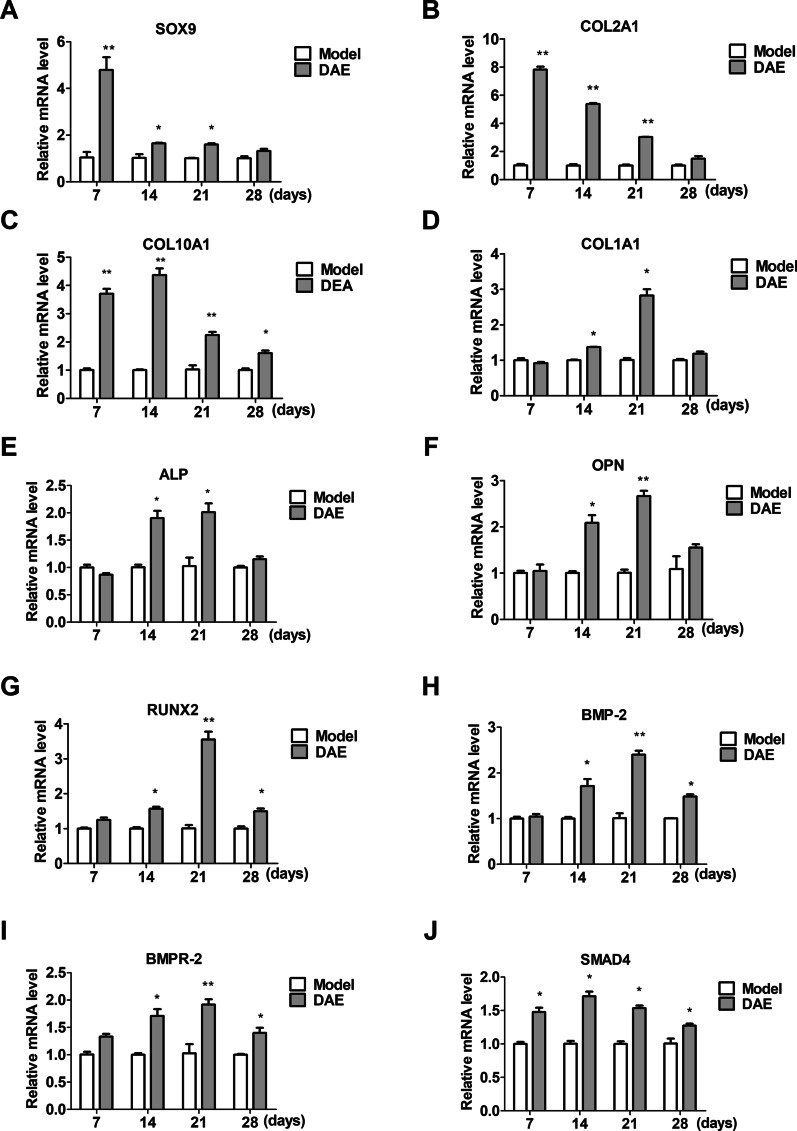
Expression of marker genes during fracture healing. **A** The expression levels of SOX9 in the model group and DAE group at 14, 21, 28 days after fracture. **B** The expression levels of COL2A1 in the model group and DAE group at 14, 21, 28 days after fracture. **C** The expression levels of COL10A1 in the model group and DAE group at 14, 21, 28 days after fracture. **D** The expression levels of COL1A1 in the model group and DAE group at 14, 21, 28 days after fracture. **E** The expression levels of ALP in the model group and DAE group at 14, 21, 28 days after fracture. **F** The expression levels of OPN in the model group and DAE group at 14, 21, 28 days after fracture. **G** The expression levels of RUNX2 in the model group and DAE group at 14, 21, 28 days after fracture. **H** The expression levels of BMP-2 in the model group and DAE group at 14, 21, 28 days after fracture. **I** The expression levels of BMPR-2 in the model group and DAE group at 14, 21, 28 days after fracture. **J** The expression levels of SMAD4 in the model group and DAE group at 14, 21, 28 days after fracture. (Model group sample data as control, *0.05 > *P* > 0.01; **0.01 > *P* > 0.001; ***P < 0.001; *n* = 3)

The gene expression analysis showed that the expression levels of SOX9 and early cartilage marker COL2A1 in the DAE treatment group were significantly increased on the 7th, 14th and 21st days (Fig. 4A–B). Meanwhile, compared with the model group, the expression levels of hypertrophic cartilage marker COL10A1 in the DAE treatment group were significantly increased on the 7th, 14th, 21st and 28th days (Fig. 4C). In addition, compared with the model group, the expression of RUNX2 in the treatment group was significantly increased on the 14th, 21st and 28th days, the expression of OPN was significantly increased on the 14th and 21st days (Fig. 4F–G), the expression of osteogenic marker COL1A1 was significantly increased on the 14th and 21st days (Fig. 4D), and the expression of bone mineralization marker ALP was significantly increased on the 14th and 21st days (Fig. 4E). Interestingly, except for the 7th day, the expressions of BMP-2 and BMPR-2 in the treatment group were significantly up-regulated (Fig. 4H–I), and SMAD4 was significantly increased on the 7th, 14th, 21st and 28th days (Fig. 4J), indicating that BMP-2/SMAD4 signal may be activated in the process of fracture healing after DAE treatment.

**Fig. 5:**
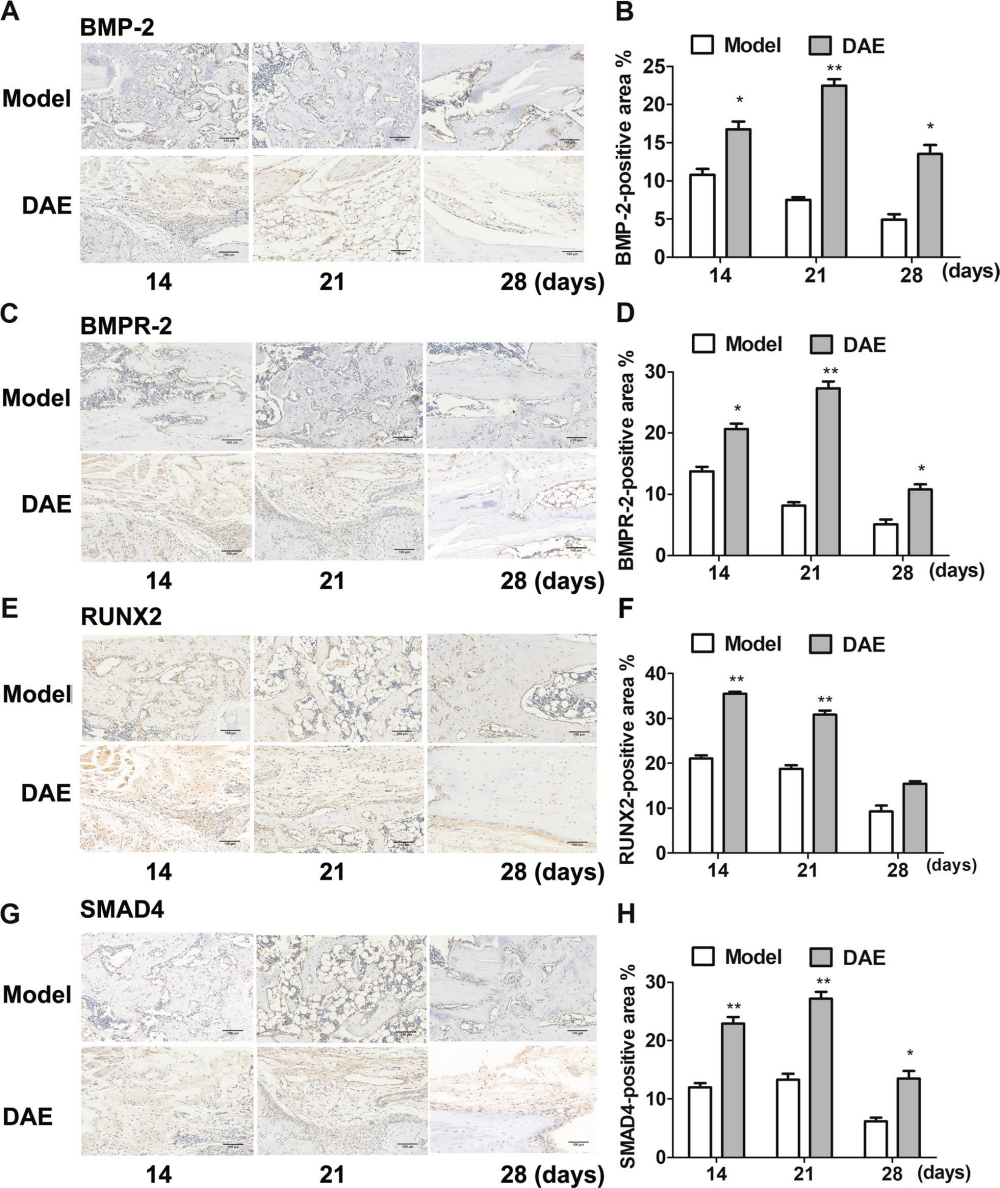
Immunohistochemical analysis of fracture zone at 14, 21 and 28 days after fracture. **A** Representative images of the fractured femurs stained for BMP-2. **B** Quantification of the positively stained area in the whole fracture callus for BMP-2. **C** Representative images of the fractured femurs stained for BMPR-2. **D** Quantification of the positively stained area in the whole fracture callus for BMPR-2. **E** Representative images of the fractured femurs stained for RUNX2. **F** Quantification of the positively stained area in the whole fracture callus for RUNX2. **G** Representative images of the fractured femurs stained for SMAD4. **H** Quantification of the positively stained area in the whole fracture callus for SMAD4. (Model group sample data as control, *0.05 > *P* > 0.01; **0.01 > *P* > 0.001; ***P < 0.001; *n* = 3)

BMP-2, BMPR-2, SMAD4 are the key molecules of BMP-2/SMAD4 signaling pathway, and RUNX2 is the key marker of osteogenesis, so we analyzed the above markers by immunohistochemistry and further detected the key role of BMP-2/ SMAD4 signaling pathway in the treatment of fracture by DAE. Compared with the model group, the protein expressions of BMP-2, BMPR-2 and SMAD4 were significantly increased on the 14th, 21st and 28th days after treatment with DAE treatment, and the protein expressions of RUNX2 were significantly increased on the 14th and 21st days after treatment (Fig. 5A–H). The expression of RUNX2 was up-regulated on day 28, but the difference was not statistically significant. Because BMP-2/SMAD4 signaling pathway plays a role in regulating osteogenesis, the up-regulation of RUNX2 in DAE group may be related to the activation of BMP-2/SMAD4 signaling pathway in DAE group. According to the above results, the fracture of DAE group was close to healing on the 28th day, so the RUNX2 situation was stable, although the difference was not statistically significant.

**Fig. 6:**
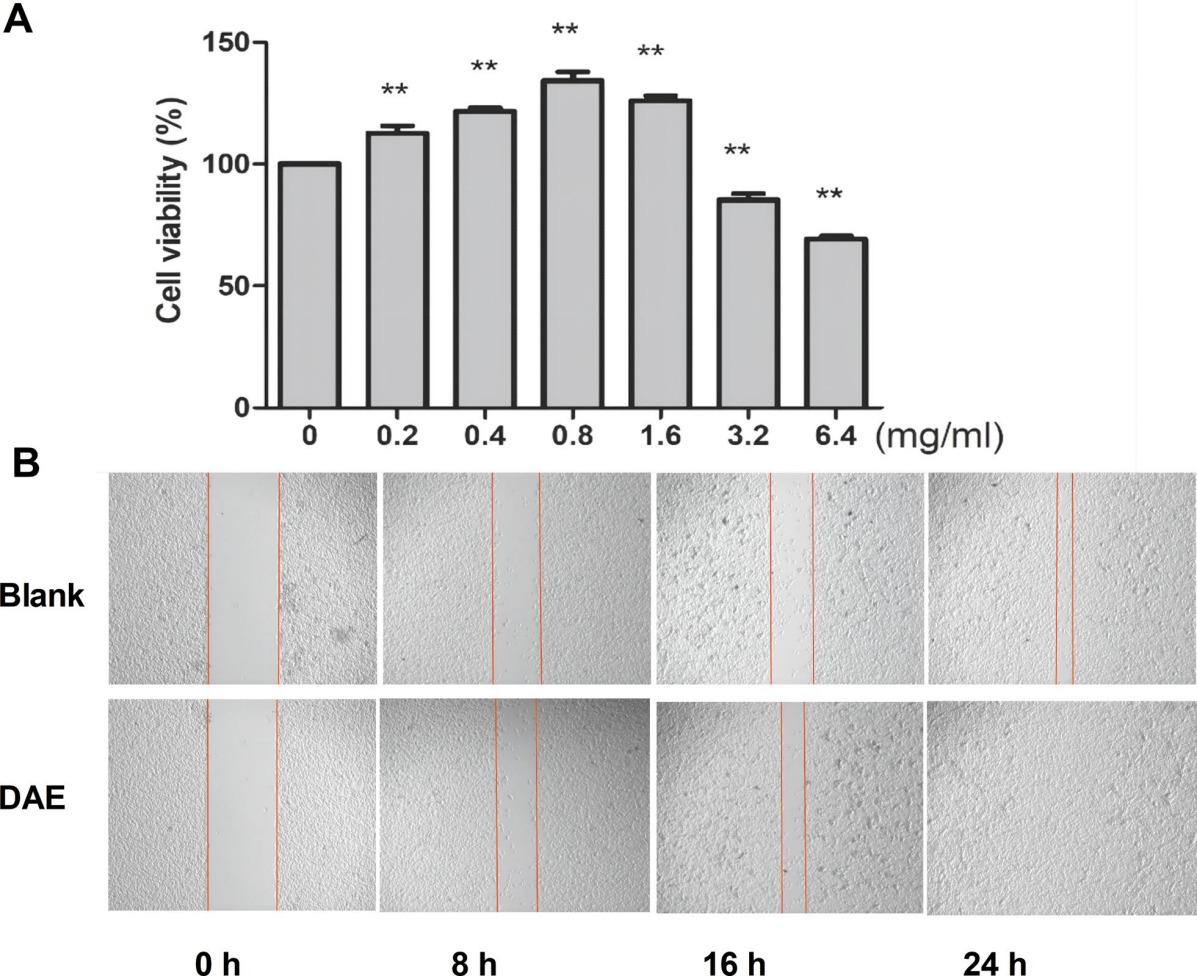
Cell viability assay and wound scratch test. **A** CCK-8 results represent the results of cell proliferation experiments. In a certain concentration range, DAE has a proliferation effect on MC3T3-E1 cells. **B** Scratch test representing cell migration ability of blank group and DAE group

CCK-8 method was used to determine the ability of cells proliferation under treatment with different concentrations of DAE. The results showed that with the increase in the concentration of DAE, the proliferation effect of MC3T3-E1 was increased, the maximum at 0.8 mg ml^−1^, and the proliferation difference was statistically significant. The proliferation of MC3T3-E1 was significantly inhibited at the concentration of 3.2 mg ml^−1^ and above, and the difference was statistically significant (Fig. 6A). The scratch test was used to examine the effect of DAE on the ability of MC3T3-E1 cell migration. It can be found that DAE at the optimal concentration can enhance the migration ability of MC3T3-E1 cells (Fig. 6B). Scratch test can simulate the internal environment of fracture in vivo. DAE promotes the migration and movement of MC3T3-E1 cells, which is very similar to the effect of DAE on tibial fracture in vivo.

## Conclusion

As a valuable traditional Chinese medicine, deer antler contains abundant proteins, peptides and oligopeptides, which are the most important bioactive component in it. Proteins in deer antler mainly include keratin, collagen, estrogen receptor and cytoskeletal protein [19] and also include various enzymes [20], such as superoxide dismutase (SOD), catalase (CAT) and casein enzyme, which play an important role in improving bone metabolism and bone nutrition.

The results of this study showed that deer antler had a positive effect on bone tissue both in vivo and in vitro. Fracture healing can be divided into inflammatory stage, soft tissue stage, hard tissue stage and remodeling stage in time, and these stages show some overlap [21]. According to the current data, DAE accelerated the cartilage ossification of fracture callus. According to the results of saffron O fixation green staining, it can be found that at each stage of the fracture, the bone area in the fracture callus of the DAE treatment group was significantly increased, and the cartilage area was significantly lower than that of the model group. On the 7th day, there was no significant change in the osteogenesis-related genes. At this time, the bone callus was still dominated by cartilage-related genes, such as SOX9, COL2A1 and COL10A1, which were mainly expressed in hypertrophic chondrocytes. These chondrocytes could further mineralization and apoptosis, the expression of these genes gradually decreases, which were related to subsequent angiogenesis and intrachondral osteogenesis [22, 23]. On the 14th and 21st days, ALP, OPN and COL1A1 were significantly up-regulated, especially RUNX2 was significantly up-regulated on the 14th, 21st and 28th days. In DAE group, COL10A1 was significantly increased on the 7th, 14th, 21st and 28th day, because COL10A1 is a marker gene of hypertrophic chondrocytes, representing the differentiation of chondrocytes, which also indirectly indicates that DAE can promote fracture healing by promoting chondrocyte ossification, especially on the 7th and 14th day. With the extension of time, cartilage tissue in callus gradually decreased, and the gap gradually narrowed. These data were consistent with the measurement and analysis of histology and tissue morphology, indicating that DAE promoted the formation of cartilage in early fracture callus and promoted the ossification of cartilage in fracture callus. In the whole process of fracture healing, BMP-2 and BMPR-2, the key genes of BMP-2/SMAD4 signaling pathway, began to increase significantly on the 14th day, and the time node of their up-regulation was the same as the time node of the up-regulation of osteogenesis-related genes. Therefore, it can be inferred that DAE mainly regulates osteogenesis through BMP-2/SMAD4 signaling pathway or promotes intrachondral ossification to promote fracture healing. BMP-2 plays an important role in chondrocyte differentiation and maturation. BMP signaling pathway is known to regulate bone and cartilage development and regeneration. As an efficient bone inducer, BMPs are the initial signal molecules for the transformation of mesenchymal cells into bone cell lines, which initiates the differentiation of mesenchymal cells into bone cells. BMP/SMAD signaling pathway is one of the most important cell signaling pathways, [11, 12], and its overall effect is the positive regulation of the body's osteogenic process. The receptor involved in BMP signaling is a transmembrane glycoprotein, mainly type I receptor and type II receptor. BMP forms a complex by binding to type II receptor. Type II receptor is activated by autophosphorylation and rapidly binds to type I receptor to form a ternary complex. At this time, under the action of activated type II receptor kinase, type I receptor is phosphorylated and activated, and then, type I receptor kinase activates signal transduction molecules R-SMADs (including SMAD1, SMAD3, SMAD5, SMAD8), so that R-SMADs are phosphorylated. After the active phosphorylated R-SMADs binds to a common regulatory signaling molecule SMAD4, the signal is transmitted to the nucleus, acting together on the target gene to transcribed and translated to express the corresponding protein [12]. In this study, SMAD4 increased on the 7th day, while BMP-2 and BMPR-2 increased significantly on the 14th day, indicating that DAE can promote the healing of fracture injury through BMP-2/SMAD4 signaling pathway. However, due to the lack of knockout of important signaling factors in the signaling pathway, further verification experiments cannot be done, which is the lack of this experiment. Further research and strengthening will be done in the future experiment to improve this part of the experiment.

## Data Availability

The datasets used and/or analyzed during the current study are available from the corresponding author on reasonable request.
